# An anatomically enhanced and clinically validated framework for lung abnormality classification using deep features and KL divergence

**DOI:** 10.1016/j.mex.2025.103348

**Published:** 2025-05-14

**Authors:** Suresh Kumar Samarla, Maragathavalli P

**Affiliations:** a IT, Puducherry Technological University, Puducherry, India; b CSE, SRKR Engineering college, Bhimavaram, Andhra Pradesh, India

**Keywords:** Anatomical segmentation, Color based enhancement, KL divergence, Deep learning, Chest X-rays, Lung Abnormality, Pneumonia Detection, Anatomical Segmentation and Color-Based Enhancement

## Abstract

Detecting lung abnormalities via chest X-rays is challenging due to understated tissue variations often ignored by traditional methods. Augmentation techniques like rotation or flipping risk distorting critical anatomical features, actually leading to misdiagnosis. This paper proposes a novel two-stage ASCE (Anatomical Segmentation and Color-Based Enhancement) framework for precise and efficient classification of lung abnormalities while preserving anatomical integrity.

Stage 1 classifies Normal vs. Pneumonia with 95 % accuracy, an AUC of 0.98, and an F1-score of 0.92. Stage 2 distinguishes Pneumonia into Viral and Bacterial subtypes with 100 % accuracy and F1-score. This approach integrates segmentation and tissue-specific color enhancements with Kullback-Leibler (KL) divergence, quantifying deviations from healthy lung regions for improved classification. The lightweight pipeline ensures computational efficiency (∼0.06s/image) and clinical interpretability by preserving diagnostic features, enhancing visibility, and enabling quantitative analysis.

1. **Preserving Anatomical Structures:** The methodology ensures that diagnostic features are preserved and highlighted with Anatomy-Preserved Segmentation
2. **Enhancing Diagnostic Visibility:** The system employs targeted colour-based enhancement that improves the visibility of potential abnormalities
3. **Quantitative Analysis with Kullback-Leibler (KL) divergence:** The model enhances precise identification of abnormal tissue by comparing the probability distributions of healthy lungs and abnormal areas

## Graphical abstract

**Figure:**
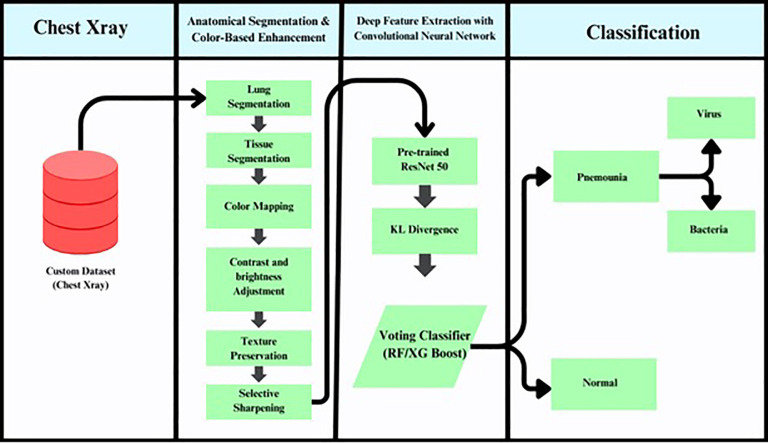
Graphical Abstract: High-level overview of the ASCE preprocessing pipeline. This figure illustrates the sequential stages involved in the Anatomical Segmentation and Color-Based Enhancement process, from lung segmentation to the application of KL Divergence for feature enhancement. Each stage is marked to highlight its contribution to enhancing the detection accuracy of lung abnormalities.

Specifications table

**Table:** 

Subject area:	Computer Science
More specific subject area:	Medical Imaging, Computer Vision, Deep learning
Name of your method:	Anatomical Segmentation and Color-Based Enhancement
Name and reference of original method:	Custom Methodology
Resource availability:	Code and Processed data will be made available on request

## Background

Lung abnormalities such as viral infections, bacterial infections, and pneumonia create substantial diagnostic problems in chest X-rays due to minor variations in tissue structure and texture. Traditional preprocessing and general augmentation procedures (e.g., rotation, flipping) often prove inadequate because they distort anatomical features, resulting in misinterpretation. Anatomical structure-preserving approaches have become increasingly important in medical imaging, particularly for detecting early-stage illnesses characterized by insignificant variations in lung tissue. This paper proposes Anatomical-Aware Color Segmentation & Preservation a method that maintains the integrity of lung structures while enhancing regions of interest and improve the detection of abnormalities. This technique combines anatomical segmentation, color-based enhancement, and quantitative abnormality detection via KL divergence to deliver a comprehensive preprocessing pipeline for lung abnormality detection.

### Related work

Many studies have aimed to improve medical image analysis by various segmentation, enhancement, and classification techniques. However, many of these approaches fall short in preserving the anatomical integrity of lung structures, which poses significant challenges in correctly detecting abnormalities. The following sections label the studies based on their primary focus areas: Image Enhancement and Preprocessing, Denoising Techniques, Feature Extraction and Selection, Classification and Retrieval Systems, and Comprehensive Reviews

#### Image enhancement and preprocessing

Hassanpour et al. (2015) explored the use of Top-hat morphological transformations to enhance the contrast of lung structures in chest X-rays. This technique demonstrated effective in highlighting abnormalities such as tumors and lung diseases by improving the visibility of fine anatomical structures. However, the study was limited in scenarios with highly overlapping structures, reducing its applicability in complex lung abnormalities [1].

Siracusano et al. (2020) proposed the PACE system (Pipeline for Advanced Contrast Enhancement, which combined FABEMD (Fast and Adaptive Bidimensional Empirical Mode Decomposition) and Contrast Limited Adaptive Histogram Equalization (CLAHE). This combination significantly enhanced image contrast, thereby improving the detection of lung lesions in chest X-rays. The study demonstrated significant improvements across three metrics: contrast improvement index, entropy, and measure of enhancement, establishing PACE as a flexible tool for advanced medical image analysis [2].

Singh et al. (2022) explored morphological filters, specifically open and bottom-hat functions, to enhance CT scan images. This enhancement improved contrast and brightness, which helps in detection of lung cancer. The study demonstrated image quality enhancement by reducing noise and enhancing diagnostic accuracy. On the other hand, the technique showed sensitivity to noise in very low-quality images, necessitating additional preprocessing steps to mitigate this limitation [3].

Karishmarao et al. (2023) presented a method using Dual-Tree Complex Wavelet Transform and adaptive morphology for improving non-contrast CT images. The approach enhances image quality by processing low and high-frequency subbands, applying denoising and local enhancement techniques. Experiments showed significant improvements in peak signal-to-noise ratio, contrast, and brightness [4]

Pavankumar et al. (2023) presented a method combining preprocessing (multidimensional filtering and histogram equalization) with segmentation and feature extraction. Using deep neural networks, this approach achieved high accuracy and significant improvements in early lung cancer detection [5].

#### Denoising techniques

Sharma et al. (2022) studied various image enhancement techniques in conjunction with deep learning algorithms for diagnosing COVID-19 from lung chest X-ray (CXR) images. The study employed pre-processing methods such as histogram equalization, CLAHE, and gamma correction, alongside filters like total variation and Gaussian denoising. Multiple Convolutional Neural Network (CNN) models, including MobileNet and DenseNet201, were tested, achieving up to 93.25 % accuracy in a 4way classification task A key limitation emphasized in the study is the need for larger datasets to strengthen the robustness and its practical effectiveness in real-world applications [6].

Abuya et al. (2023) presented a novel denoising technique for CT scan images that mixes anisotropic Gaussian filters, wavelet transforms, and a deep convolutional neural network (DnCNN). This method focused on eliminating additive Gaussian blur noise while preserving complex image details. The results indicated an average Peak Signal to Noise Ratio of 28.28 and a similarity index near 1.0, showcasing important enhancements in image quality. However, the approach was hampered by high computational complexity and the necessity for further refinement to facilitate real-time applications [7].

#### Feature extraction and selection

Singh et al. (2022) engrossed on feature extraction techniques, including Histogram Oriented Gradients based Bag of Visual Words, wavelet transforms, Local Binary Patterns, Scale-Invariant Feature Transform, and Zernike moments. To improve feature selection, the Particle Swarm Optimization algorithm was employed, suggestively enhancing segmentation performance using the Fuzzy C-Means algorithm. The Extreme Learning Machine classifier outperformed other classifiers based on metrics such as classification accuracy, error rate, precision, recall, and F-measure [8]. Aktas et al.(2023) introduced a method in which CNN-based architectures were used for feature extraction of the texture from chest X-ray images in the case of pneumonia and other lung diseases. The work showed that deep learning methods are outstandingly good at capturing the fine-grained variations between the diseased and healthy lungs, especially in cases where the dataset shows low inter-class variance, hence an accuracy of 84.57 % for binary classifications of normal and abnormal chest X-rays [9].

#### Classification and retrieval systems

Souid et al.(2021) proposed MobileNet V2 for classifying lung abnormalities using transfer learning and metadata. With an average AUC of 0.811 and over 90 % accuracy, the method was suitable for low-computation environments, such as IoT devices. Future research will explore alternative model architectures and the integration of segmentation information for clinical efficiency evaluation [10].

Kim et al. (2022) used EfficientNet v2-M for classifying lung diseases, including pneumonia and pneumothorax, achieving high validation accuracy and specificity. The study emphasized data augmentation and hyperparameter tuning for effective multi-class classification. Challenges were identified in multi-class classification, particularly balancing dropout regularization to avoid overfitting while preserving meaningful information. Optimally tuning hyperparameters improved model performance, showing promise for multi-class lung disease diagnosis [11].

Chokchaithanakul et al. (2022) introduced the Lung Balance Contrast Enhancement Technique to mitigate dataset mismatch in chest radiography. The method normalized lung regions and applied targeted augmentations. The study achieved the highest AUC scores for tuberculosis detection in out-of-domain datasets. the system also improves heatmap visualizations, though lower scores indicate room for improvement. Future work aims to validate this method on other conditions like pneumonia and COVID-19 [12].

Agrawalet al. (2022) developed a Content-Based Medical Image Retrieval system aimed at classifying and retrieving lung disease-related X-ray images. Leveraging deep learning models with transfer learning, the system achieved a 49.71 % improvement in precision and a 26.55 % increase in the Area Under Precision-Recall Curve across various distance metrics. Tested on COVID-19 X-ray datasets, the system attained an accuracy of 81 % through 5fold cross-validation. Future work suggested expanding the dataset, incorporating more subclasses, and optimizing the system with additional deep learning models [13].

Suresh et al.(2024) proposed a modified CNN model, using transfer learning to precisely diagnose lung anomalies. The model identified abnormalities with 92.18 % accuracy and varying success rates in illnesses such as cardiomegaly, pneumonia, lung opacity, and pleural effusion, demonstrating its potential in telemedicine [14].

Siddiqi et al. (2024) reviewed the deep learning algorithms for pneumonia detection using chest Xrays, particularly Vision Transformers (ViTs), However, challenges like dataset biases, class imbalance, and adversarial attacks need further research [15].

Vinayak Sharma et al. (2024) developed deeplearning-based models to detect tuberculosis from chest X-ray images. They trained a UNet segmentation model on X-ray data from the Montgomery and Shenzhen datasets, achieving an accuracy of 96.35 %. Next, the segmented lung regions from the NIAID TB dataset were classified using the Xception model, achieving 99.29 % accuracy. The study also used Grad-CAM heatmaps to visualize TB lesions. Variations in X-ray machine output quality may have an impact on the AI system’s outcomes. In addition, larger and more diversified datasets are required, which need the use of high-performance GPUs or supercomputers, resulting in higher prices and longer training durations. Noise in X-ray images may also have an impact on system performance, however this can be addressed through denoising or color normalization. Finally, AI systems require institutional approval for medical use, which limits their utility as main diagnostic tools [16].

M V Sanidia et al. (2024) demonstrates a deep learning architecture for multi-class detction of lung abnormalities like viral pneumonia, fibrosis, tuberculosis, opacity, and COVID-19, using chest X-rays. The model uses a proprietary convolutional neural network (CNN) to improve feature maps for discriminative learning. When tested on a large-scale dataset, it obtained 98.88 % accuracy with an AUC of 0.9939. Future research seeks to use GANs to generate synthetic medical images, enhance generalization across patient demographics, and link them with other diagnostic tools to provide more thorough assessments [17].

Suresh kumar et al. (2024) presents a novel approach for classifying lung abnormalities using a 3M-CNN and an early fusion technique, trained on the CheXpert dataset. The ensemble method, which fuses sub-model outputs, demonstrated high accuracy for conditions like Pneumothorax but struggles with abnormalities such as Enlarged Cardiomediastinum and Fracture. Despite its strong overall performance, further refinement is necessary for specific conditions to enhance diagnostic precision and accuracy [18].

Francis et. al used Densely Connected Convolutional Neural Network (DCNN), to detect Pneumonia, and COVID-19 in chest Xrays. From the results, the performance to parameter size ratio highlighted this method's effectiveness to train a DenseNet model with lesser parameters compared to existing Deep CNN (DCNN) models, yet yield promising results [19].

#### Comprehensive reviews

Xiaoyan Jiang et al. (2023) provided a comprehensive review of deep learning applications in cancer diagnosis using various medical imaging techniques, including X-rays, CT and MRI. The review covered fundamental deep learning architectures, traditional pretrained models, and innovative neural networks such as transfer learning, ensemble learning, graph neural networks, and Vision Transformers. Additionally, it summarized overfitting prevention methods like dropout, batch normalization, weight initialization, and data augmentation. Despite the fact deep learning has achieved notable success in image classification, reconstruction, detection, segmentation, registration, and synthesis, challenges continue. These include the insufficiency of highquality labeled datasets, complications in rare cancer diagnosis, multi-modal image fusion, and problems related to model explainability and generalization. The review emphasized the need for powerful preprocessing mechanisms, more public standard datasets, improved pre-training models, and focused research on multimodal data fusion and supervised paradigms to advance the field further [20].

#### Machine learning for lung cancer detection

Mohammad Mustafa et al. (2024) proposed a CNNbased model to classify lung cancer by Preprocessing and balancing the dataset with SMOTE, resulted in high classification accuracy, high precision, recall, and F1-scores The model emphasizes early detection and non-invasive diagnosis. Future research should focus on external validation, model interpretability, and potential applications for other cancers or medical imaging [21]

#### Comparative studies

The collective studies highlight a drift towards integrating advanced image processing with deep learning to enhance the quality and diagnostic utility of medical images. Morphological transformations and filters are commonly employed for initial image enhancement, improving contrast and reducing noise, which are critical for accurate disease detection. Denoising techniques, particularly those leveraging deep convolutional neural networks, have shown promise in preserving image details while removing noise, albeit with challenges related to computational demands. Feature extraction and selection remain critical for optimizing model performance. Classification and retrieval systems improve significant advantages from transfer learning and deep CNN architectures, achieving high accuracy and enhanced retrieval metrics. Comprehensive reviews underscore the need for powerful preprocessing mechanisms, large and high-quality datasets and the exploration of emerging deep learning models to address current limitations.

### Comparison with existing studies

Advancements in deep learning for lung abnormality detection have emphasized the critical need for image preprocessing techniques that do not compromise the anatomical integrity of lung structures. Current preprocessing methods often modify crucial medical features, thereby affecting diagnostic accuracy. Our system, the Anatomical Segmentation and Color-Based Enhancement (ASCE), builds upon the foundational image processing techniques used by Hassanpour et al. [1] and integrates advanced neural network architectures similar to those proposed by Pavankumar et al. [3]. This integration allows ASCE to maintain the integrity of lung tissues in X-rays effectively, which is crucial for accurate diagnoses.

ASCE significantly advances the capabilities of standard methods, which often struggle with overlapping structures or noise sensitivity. By employing specific edge detection and color-based segmentation, ASCE enhances the visibility of lung tissues, thus improving the accuracy of both segmentation and classification of lung abnormalities. This method not only addresses the common limitations observed in other preprocessing techniques but also ensures that enhancements are tailored specifically to lung structures, thereby improving diagnostic reliability in real-time applications.

For a detailed comparison of how ASCE stands in relation to other studies, including the efficacy and limitations of each method was presented in Table 1. This table highlights the distinct advantages of ASCE over traditional techniques such as Top-Hat transformations [1], PACE systems [2], and various neural network applications [[4], [5], [6]], showcasing its superior capability to enhance diagnostic precision and handle complex scenarios without the typical risks of overspecialization.

**Table 1:** Comparative Table.

Study /Method	Key Features	Effectiveness (Results)	Limitations
[1]	Top-Hat transformations for lung X-rays.	Enhances contrast.	Sensitivity to noise and over-enhancement may lead to possible misinterpretation of clinical data.
[2]	PACE system combining FABEMD with CLAHE.	Contrast enhancement and reduced brightness inhomogeneities.	Limited to COVID-19 diagnosis.
[4]	Dual-Tree Complex Wavelet Transform for CT images.	Improved SNR, contrast, and brightness.	Complex computations, potential for overfitting specific noise patterns.
[5]	Deep neural networks for early lung cancer detection.	High accuracy in early detection.	Dependent on the quality of input data, requires extensive computational resources.
[6]	Deep learning with histogram equalization for COVID-19 CXR.	93.25 % accuracy in 4-way classification.	Computationally intensive, risk of misclassification in unseen data
Proposed Method	Anatomical Segmentation and Color-Based Enhancement	Presumed to enhance diagnostic precision by improving image segmentation and colour enhancement in relevant anatomical structures.	Risk of overspecialization to anatomical features.

## Method details

The proposed methodology Anatomical Segmentation and Color-Based Enhancement (ASCE) begins with data preparation, The dataset was collected from publicly available data sources [22] and organized into three folders: one for chest X-ray images, another for annotations, and a third for lung masks. Each folder contains data categorized into 3 classes: normal (916 images), virus (1401 images), bacteria (2587 images). To guarantee data integrity and consistency, each sample undergoes validation to check the presence of all required components: images, annotations, and masks. We used 80 % for training, 10 % for validation, and 10 % for testing. The ASCE algorithm (Algorithm 1 and Fig. 3) presents the process followed in detection of lung abnormalities by combining segmentation, enhancement, feature extraction and statistical analysis.

**Algorithm 1:** Lung Abnormality Classification using Anatomical Segmentation and Color-Based Enhancement (ASCE) Technique.

1: **Input:** Chest X-ray images, masks, annotations
2: **Output:** Classification of lung abnormalities (normal, pneumonia, then virus, bacteria)
3: **Step 1: Data Preparation**
4: Organize chest X-ray images, segmentation masks, and annotations.
5: Load image files, masks, and annotation files.
6: Verify that all files (image, mask, annotation) are present for each sample.
7: **Step 2: Anatomical Segmentation and Color-Based Enhancement**
8: Perform lung segmentation by applying the lung mask to each X-ray image.
9: Perform tissue segmentation by thresholding the segmented lung region.
10: Apply color-based enhancement to improve the visibility of abnormalities in the segmented image.
11: Generate an enhanced image using the method.
12: **Step 3: Feature Extraction with** ResNet50
13: Pass the enhanced images through a pre-trained ResNet50 model to extract features F.
14: Calculate the KL Divergence between the original and enhanced healthy references vector (Q):
$D_{KL}\left(P|Q\right)=\sum_{i}P\left(i\right)\log\left(\frac{P(i)}{Q(i)}\right)$
15: Append KL to Feature Vector F’: features = np.append(F, KL)
16: **Step 4: Classification:** Pass F' to Voting_Classifier (RF + XGBoost)
17: Predict class based on soft-voting scores
18: **Step 5: Evaluation and Visualization**
23: Evaluate the model using accuracy, recall, precision, F1-score, BA, MCC and Cohen’s Kappa.
25: Analyze the confusion matrix for misclassification patterns.

**Fig. 1:**
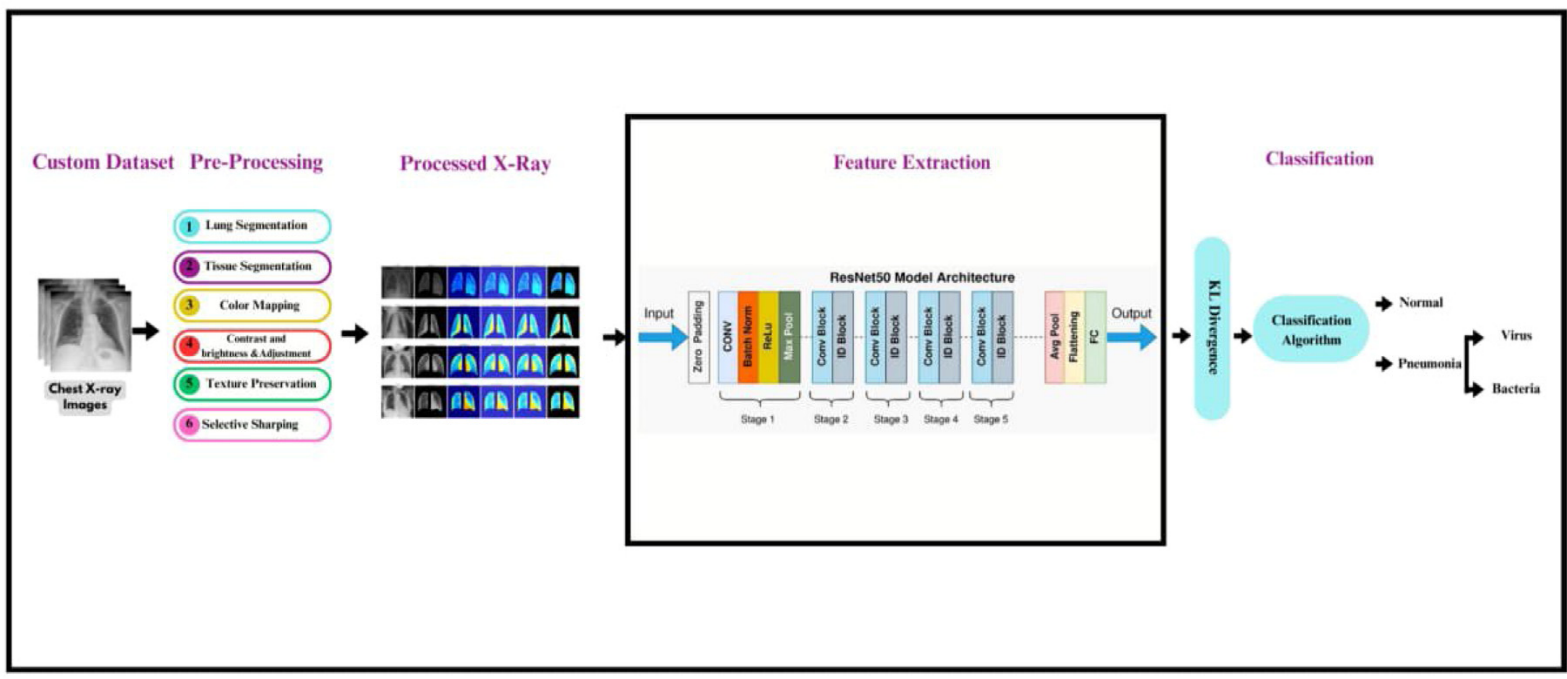
Comprehensive Workflow for Chest X-Ray Analysis from Preprocessing to Classification. This diagram illustrates the sequence of steps applied to chest X-ray datasets, including lung and tissue segmentation, colour mapping, and texture preservation, followed by feature extraction using the ResNet50 architecture, concluding in the classification of images into categories such as Pneumonia, and Normal and then Viru vs Bacteria.

The core of the method lies in the ASCE (Anatomical Segmentation and Color-Based Enhancement). the work flow from preprocessing to classification is presented in Fig. 1, lung segmentation is performed by applying the segmentation mask to the X-ray images to isolate the lung region. Then, tissue segmentation is carried out using thresholding, which highlights the abnormal tissues. To further enhance the image, color-based brightness and contrast adjustments are applied. The final result is a **lung-only, tissue-enhanced color X-ray**, then followed by **feature extraction**, where each enhanced image is passed through a pretrained ResNet-50 (with the classification head removed), extracting feature vectors from the final convolutional layer. These deep features characterize the global and local patterns retained after ASCE processing. **SMOTE** algorithm is employed to synthetically balance the training data before classification. A **two-stage approach** is used for classification:

• **Stage 1:** A soft-voting ensemble of **Random Forest** and **XGBoost** classifiers distinguishes between *normal* and *Pnemonia* cases.
• **Stage 2:** On images identified as Pnemonia, a second XGBoost classifier determines the specific pathology — *viral* or *bacterial* pneumonia.

Furthermore, **Kullback–Leibler (KL) divergence** is calculated between the class probability distributions of the RF and XGB models to estimate classifier disagreement. A low KL value shows model confidence and higher is treated as a signal for indeterminate or borderline cases.

The final model is assessed using a comprehensive set of metrics including accuracy, precision, recall, F1-score, AUC, balanced accuracy, MCC, and Cohen’s kappa. Confusion matrix plots, ROC and PR curves, and KL divergence analysis additional support interpretability and error analysis.

Fig. 1**illustrates the full ASCE method pipeline**, representing how raw input (chest X-rays) undergoes transformation through preprocessing, segmentation, color-based enhancement, and classification steps. This structured frame work emphasizes the method’s uniqueness and completeness by merging **deep learning, statistical confidence (KL divergence)**, and **conventional ensemble classifier.**

## Mathematical background for image processing pipeline

### Stage 1: anatomical segmentation

The main goal of this is the segmentation of lung regions from chest X-ray images, thereby enabling further processing and enhancement. It includes two steps: one, lungs segmentation from chest X-rays; two, tissue segmentation from lungs.

#### Lung segmentation

The lung segmentation process often uses a convolutional neural network. The network applies a series of convolutional layers with a non-linear activation function (ReLU) to extract features from the input Xrays. The final layer outputs a probability map P(x,y), where each pixel denotes the probability that the matching pixel of the lung region

(1) $$f\left(x,y\right)=\sum_{i=1}^{k}\sum_{j=1}^{k}I\left(x+i,y+j\right)\cdot K\left(i,j\right)$$

where f(x,y) is the output feature map, K(i,j) is the convolution kernel (filter) and I(x,y) is the input image.

The lung mask M(x,y) is then generated by applying a threshold T on the probability map:

(2) $$M\left(x,\;y\right)\;=\left\{ \begin{array}{c} 1,\;if\;P\left(x,\;y\right)\;>\;T\\ 0,\;otherwise \end{array}\right.$$

#### Tissue segmentation within lungs

Similar to lung segmentation, tissue segmentation uses a CNN to classify each pixel within the lung region into different tissue types (e.g., bronchi, alveoli, blood vessels). The model outputs multiple probability maps, one for each tissue type. Softmax function is used to ensure that the output for each pixel across all classes sums to 1.

(3) $$S_{i}\left(x,y\right)=\frac{e^{z_{i}\left(x,y\right)}}{\sum_{j=1}^{N}e^{z_{j}\left(x,y\right)}}$$

where z_i_ is the input to the softmax function for the i th class (tissue type), and S_i_ is the probability of the pixel belonging to that class.

Labeled tissue regions are then assigned based on the maximum probability:

(4) $$L\left(x,y\right)=\arg\max_{i}S_{i}\left(x,y\right)$$

where L(x,y) is the label of the tissue type for the pixel at (x,y).

### Stage 2: color-based enhancement

This stage allows for differentiating various lung structures such as alveoli, bronchi, and blood vessels.

#### Color mapping

Color mapping involves assigning specific RGB color values to different tissue types based on their labels. This is a straightforward mapping where each label corresponds to a specific color. Given a label L(x,y), the color-mapped image C(x,y) can be defined as:

(5) C(x,y)=color_map[L(x,y)]

where color map is a dictionary mapping labels to RGB values

#### Contrast and brightness adjustment

Contrast and brightness adjustments are linear operations applied to the image pixels. Contrast adjustment scales the pixel intensities, while brightness adjustment shifts them.

(6) I(x,y)=α·I(x,y)+β

where I(x,y) is the original pixel intensity, α is the contrast factor, β is the brightness offset, and I′(x,y) is the adjusted pixel intensity.

#### Texture preservation

Texture preservation uses techniques like bilateral filtering, which smooth out the image while conserving edges as both spatial distance and intensity difference.

(7) $$I^{\prime }\left(x,y\right)=\frac{1}{W_{p}}\sum_{x_{i},y_{i}}I\left(x_{i},y_{i}\right)\cdot f_{d}\left(\left|\left|\left(x,y\right)-\left(x_{i},y_{i}\right)\right|\right|\right)\cdot f_{r}\left(\left|I\left(x,y\right)-I\left(x_{i},y_{i}\right)\right|\right)$$

(8) $$W_{p}=\sum_{x_{i},y_{i}}f_{d}\left(\left|\left|\left(x,y\right)-\left(x_{i},y_{i}\right)\right|\right|\right)\cdot f_{r}\left(\left|I\left(x,y\right)-I\left(x_{i},y_{i}\right)\right|\right)$$

➢ (I'(x,y)) is the filtered intensity at pixel ((x,y)).
➢ (I(x_i, y_i)) is the intensity of neighboring pixels.
➢ (f_d_(||(x,y) - (x_i_, y_i_)||)) is the spatial weighting function (Gaussian function based on distance).
➢ (f_r_ (|I(x,y) - I(x_i_, y_i_)|)) is the range weighting function (Gaussian function based on intensity difference).
➢ (W_p_) is the normalization factor.

#### Selective sharpening

Selective sharpening enhances edges by applying an unsharp mask, which subtracts a unclear version of the image from the original.

(9) $$I^{\prime }\left(x,y\right)=I\left(x,y\right)+\lambda \cdot \left(I\left(x,y\right)-I_{b}\left(x,y\right)\right)$$

where I_b_(x,y) is the blurred version of the image, λ is the sharpening strength, and I′(x,y) is the improved image.

#### KL divergence

KL Divergence Ensures better separation between normal and abnormal regions. It Enhances feature extraction by minimizing overlap in probability distributions.

(10) $$D_{KL}\left(P|Q\right)=\sum_{i}P\left(i\right)\log\left(\frac{P\left(i\right)}{Q\left(i\right)}\right)$$

where P is the probability distribution for normal lungs, and Q is the distribution for abnormal lungs.

The goal of this technique is to reduce false positives and false negatives by highlighting abnormalities that may be missed with standard preprocessing methods

Convolutional neural networks (CNNs) like ResNet50 excel at extracting deep features (2048- dimensional feature vectors) from medical images but lack the ability to explicitly quantify how much an image departs from a healthy baseline. To address this limitation, KL Divergence is integrated as a statistical metric, providing a scalar score that summarizes deviations in intensity and texture distributions across lung regions. This scalar complements the deep features by offering a quantitative measure of abnormality.

Incorporating KL Divergence into the feature vector enhances the ensemble classifier's ability to discriminate pathological cases, particularly in ambiguous circumstances where ResNet50 features alone may not provide clear separability. Experimental validation demonstrates that this method significantly improves class differentiation. For example, viral pneumonia consistently shows higher KL values (KL > 10) compared to normal cases (KL < 7), underscoring the metric’s efficiency in refining model predictions and boosting overall classification performance.

**Fig. 2:**
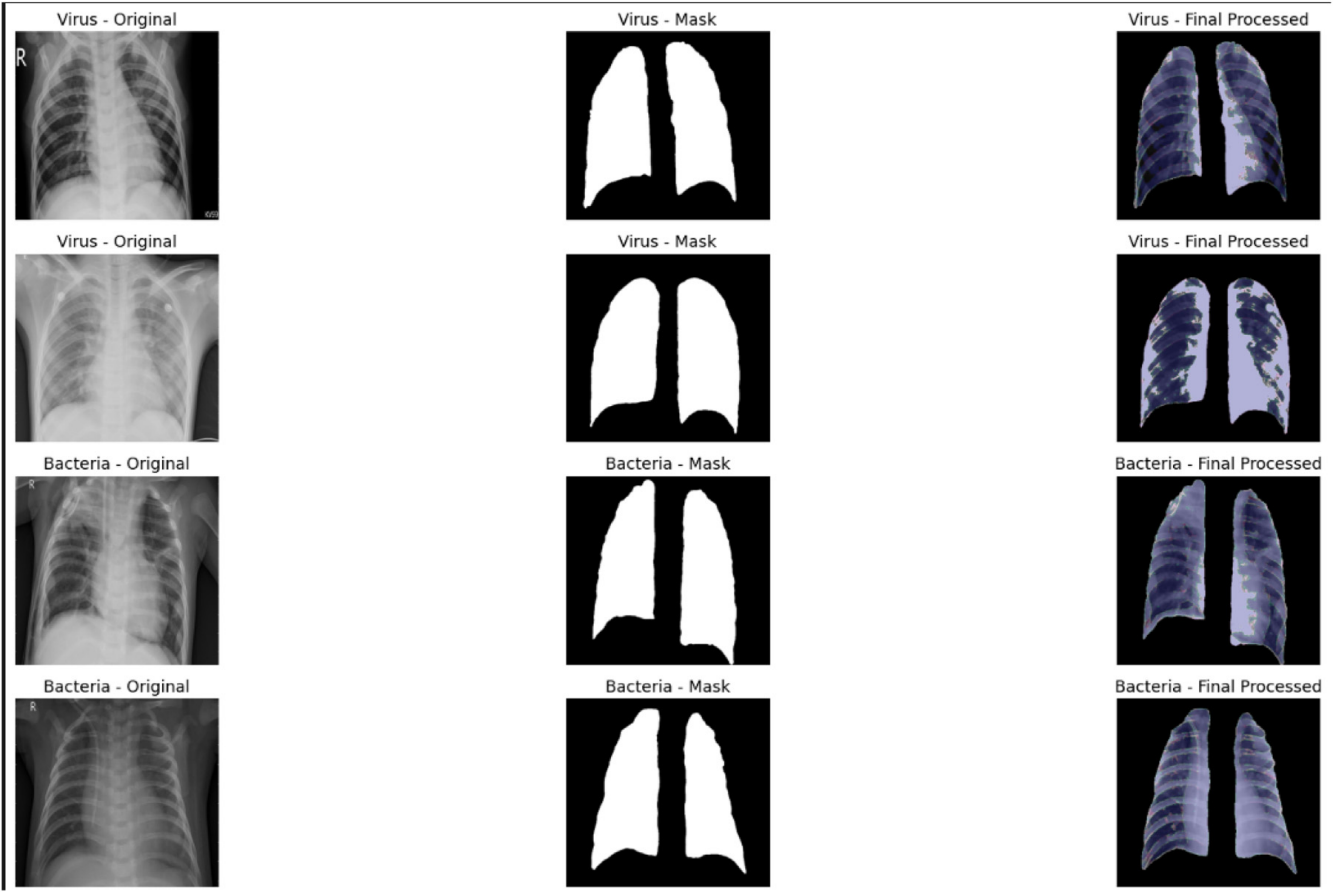
Visual plan of the ASCE preprocessing pipeline. The left column shows the original chest X-ray images, the center column displays the lung masks generated through anatomical segmentation, and the right column depicts the final enhanced output after applying tissue-specific color mapping and structural sharpening. This enhancement improves the visual separability of lung tissues such as alveoli and blood vessels, facilitating better feature extraction.

The effectiveness of anatomical segmentation and tissue enhancement is presented in Fig. 2. ASCE not only separates the lung field but also emphasizes structural differences using contrast and color-coding. This results in more discriminative feature vectors for downstream classification. The clearer visual separation of lung substructures allows both the classifier and KL divergence to more effectively distinguish between normal, viral, and bacterial patterns, especially in challenging cases like bacterial pneumonia.

**Fig. 3:**
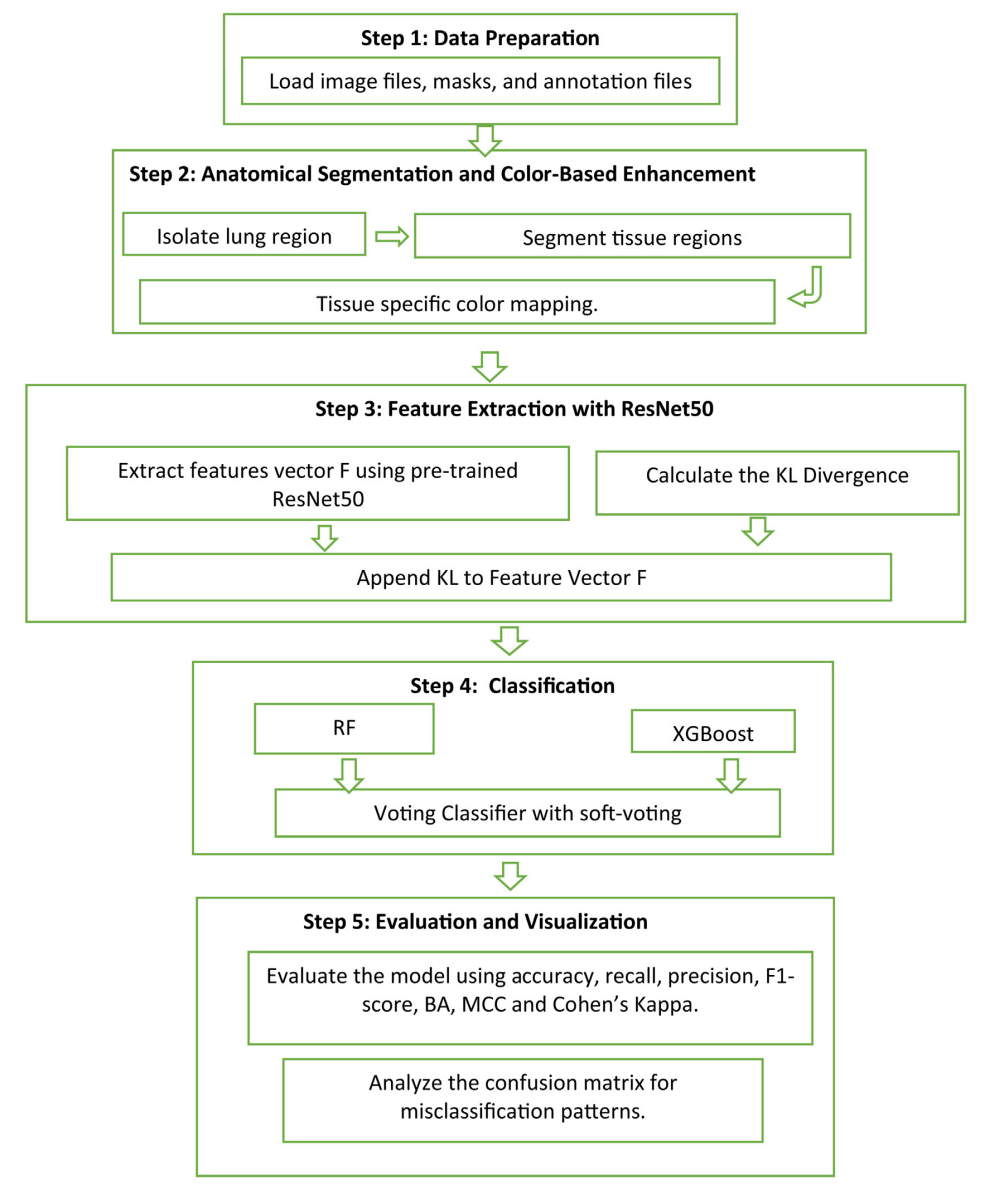
Workflow of the proposed ASCE (Anatomical Segmentation and Colour-Based Enhancement) framework. The pipeline consists of five major stages: (1) Data Preparation, including validation of image-mask-annotation pairs; (2) Anatomical Segmentation and tissue-specific Colour-Based Enhancement to highlight alveoli, bronchi, and blood vessels; (3) Feature Extraction using ResNet50 followed by KL divergence computation against a healthy reference vector; (4) Classification using a soft-voting ensemble (Random Forest + XGBoost) that incorporates both deep features and KL divergence; and (5) Evaluation using standard performance metrics.

## Performance metrics

The evaluation of model performance is based on several key metrics, including precision, recall, F1score, and accuracy. These metrics are formally defined as follows:

**Precision** measures how many of the predicted positives are actual positives:

(11) $$\text{Precision}\;=\;\frac{\text{TP}\;}{\left(\text{TP}+\text{FP}\right)}$$

**Recall** (Sensitivity) measures how many actual positives are correctly identified:

(12) $$\text{Recall}=\;\frac{\text{TP}\;}{\left(\text{TP}+\text{FN}\right)}$$

**F1-score** is the harmonic mean of precision and recall, balancing both metrics:

(13) $$F1\;\text{score}=2\;\frac{\text{precision}*\;\text{Recall}\;}{\text{precision}+\;\text{Recall}\;}$$

**Accuracy** represents the overall proportion of correct predictions:

(14) $$\text{Accuracy}=\;\frac{\left(\text{TP}\;+\;\text{TN}\right)}{\left(\text{TP}\;+\;\text{TN}\;+\;\text{FP}\;+\;\text{FN}\right)}$$

**Balanced Accuracy (BA)** accounts for imbalanced datasets by similarly weighting the true positive rate (sensitivity) and true negative rate (specificity). It is computed as:

(15) $$\text{Balanced}\;\text{Accuracy}\;(\text{BA})=\frac{1}{2}\left(\frac{TP}{TP+FN}+\frac{TN}{TN+FP}\right)$$

**Matthews Correlation Coefficient (MCC)** is a strong metric that considers all four elements of the confusion matrix (TP, TN, FP, FN) and returns a value between −1 and +1

(16) $$\text{MCC}=\frac{TP\cdot TN-FP\cdot FN}{\sqrt{\left(TP+FP\right)\left(TP+FN\right)\left(TN+FP\right)\left(TN+FN\right)}}$$

An MCC of +1 shows perfect prediction, 0 means no better than random, and −1 indicates total disagreement between prediction and ground truth values.

**Cohen’s Kappa (κ)** measures the agreement between predicted and true labels, adjusted for agreement occurring by chance:

(17) $$\kappa =\frac{p_{o}-p_{e}}{1-p_{e}}$$

Where p_o_​ is the observed agreement and p_e_​ is the expected agreement by chance. Kappa values close to 1 indicate strong agreement, while values ≤ 0 suggest poor or no agreement

### Performance evaluation and analysis of the proposed model

Table 2 presents results of our iterative refinements at each stage of the pipeline led to consistent improvements in classification performance across all abnormality classes.

1. **Segmentation Upgrade:**
  Switching from Otsu thresholding to gradient-based segmentation notably improved feature quality, mostly for complex pathological structures such as bacterial infections. The F1-score for the bacteria class enhanced from 0.46 to 0.78, validating that gradient-based methods preserved finer anatomical details, which are often critical for distinguishing overlapping lung abnormalities.
2. **Feature Extraction Advancement:**
  The transition from hand-crafted Local Binary Patterns (LBP) to deep feature extraction using ResNet50 substantially improved performance for virus and bacteria classes. The virus F1-score improved from 0.75 to 0.81, and bacteria from 0.77 to 0.81, due to ResNet50’s ability to capture hierarchical features that account for both structural texture and intensity variations.
3. **Class Balancing via SMOTE:**
  Application of SMOTE to mitigate class imbalance had a marked impact on minority class performance. Both virus and bacteria F1-scores improved from 0.77 to 0.83 and 0.78 to 0.83, respectively, by enabling the classifier to absorb more representative decision boundaries for imbalanced classes.
4. **Hyperparameter Optimization Gains:**
  Optimization using Bayesian search surpassed grid search, especially for the virus class where the F1-score rose from 0.58 to 0.82. Bayesian optimization effectively explored better parameter regions, resulting in higher recall and more stable predictions.

**Table 2:** Performance comparison of classification models with different preprocessing and feature extraction methods for lung abnormality detection.

Model	Segmentation	Feature extraction	Abnormality Class	Accuracy	Precision	Recall	F1-Score	Support
Random Forest	ASCE with Otsu Thresholding	ResNet50	Normal	0.76	0.99	1.0	1.0	354
			Virus		1.0	0.39	0.56	28
			Bacteria		0.63	0.24	0.35	302
XGBoost	ASCE with Otsu Thresholding	ResNet50	Normal	0.83	1.0	1.0	1.0	532
			Virus		0.98	0.92	0.95	148
			Bacteria		0.64	0.36	0.46	286
XGBoost (with Augmentation)	ASCE with Otsu Thresholding	ResNet50	Normal	0.83	0.97	0.96	0.96	1102
			Virus		1.0	0.32	0.49	28
			Bacteria		0.62	0.36	0.45	285
Random Forest	ASCE with Gradient Thresholding	LBP	Normal	0.86	0.93	0.91	0.92	531
			Virus		0.79	0.7	0.75	506
			Bacteria		0.73	0.81	0.77	518
XGBoost	ASCE with Gradient Thresholding	LBP	Normal	0.87	0.92	0.94	0.93	531
			Virus		0.84	0.71	0.77	506
			Bacteria		0.73	0.83	0.78	518
Random Forest (with SMOTE)	ASCE with Gradient Thresholding	ResNet50	Normal	0.89	0.93	0.96	0.94	517
			Virus		0.83	0.8	0.81	518
			Bacteria		0.81	0.81	0.81	517
XGBoost (with SMOTE)	ASCE with Gradient Thresholding	ResNet50	Normal	0.90	0.95	0.95	0.95	517
			Virus		0.85	0.8	0.83	518
			Bacteria		0.8	0.85	0.83	517
Random Forest (Grid Search)	ASCE with Gradient Thresholding	ResNet50	Normal	0.87	0.94	0.95	0.95	517
			Virus		0.83	0.82	0.83	518
			Bacteria		0.83	0.83	0.83	518
XGBoost (Grid Search)	ASCE with Gradient Thresholding	ResNet50	Normal	0.75	0.79	0.82	0.8	183
			Virus		0.69	0.51	0.58	280
			Bacteria		0.75	0.85	0.8	518
Random Forest (Bayesian Optimization)	ASCE with Gradient Thresholding	ResNet50	Normal	0.90	0.93	0.96	0.95	517
			Virus		0.86	0.77	0.81	518
			Bacteria		0.81	0.87	0.84	517
XGBoost (Bayesian Optimization)	ASCE with Gradient Thresholding	ResNet50	Normal	0.90	0.96	0.96	0.96	517
			Virus		0.84	0.8	0.82	518
			Bacteria		0.8	0.84	0.82	517
Ensemble Model	ASCE with Gradient Thresholding	ResNet50	Normal	0.91	0.96	0.96	0.96	517
			Virus		0.87	0.82	0.84	518
			Bacteria		0.82	0.86	0.84	517
**Proposed (2-Stage Ensemble + SMOTE)**	**ASCE with Gradient Thresholding**	**ResNet50**	**Normal**	0.95	**0.86**	**0.87**	**0.86**	**183**
			**Pneumonia**		**0.97**	**0.97**	**0.97**	**798**
			**Virus**	1.00	**1.00**	**1.00**	**1.00**	**257**
			**Bacteria**		**1.00**	**1.00**	**1.00**	**539**

The effectiveness of the ASCE framework with two-stage classification was assessed through rigorous experimentation, measuring both **classification performance** and **computational efficiency**. The evaluation included a range of metrics such as accuracy, precision, recall, F1-score, confusion matrix, AUC, MCC, Balanced Accuracy, and Cohen’s Kappa.

Overall classification performance of the proposed two stage framework is as follows:

**Stage 1 – Normal vs Pneumonia Classification** is highlighted in Table 3. The first stage employed a SMOTE-balanced ensemble classifier (Random Forest + XGBoost) to differentiate between *normal* and *pneumonia* lung X-rays. The model achieved **95 % accuracy**, with macro-averaged precision, recall, and F1-score above **0.91**, reflecting balanced performance across both classes.

**Table 3:** Performance Metrics for Stage 1: Binary Classification (Normal vs. Pneumonia).

Metric	Normal	Pneumonia	Macro Avg	Weighted Avg
**Precision**	0.86	0.97	0.91	0.95
**Recall**	0.87	0.97	0.92	0.95
**F1-Score**	0.86	0.97	0.92	0.95
**Accuracy**	**0.95**			
**AUC**	**0.9801**			
**MCC**	**0.8328**			
**Balanced Accuracy**	**0.9181**			
**Training Time**	**58.47 s**			
**Inference Time**	**0.06 sec**			

The **high AUC (0.9801)** and **Balanced Accuracy (0.9181)** confirm that the model is robust against class imbalance, while a strong **Matthews Correlation Coefficient (MCC = 0.8328)** affirms its reliability.

**Fig. 4:**
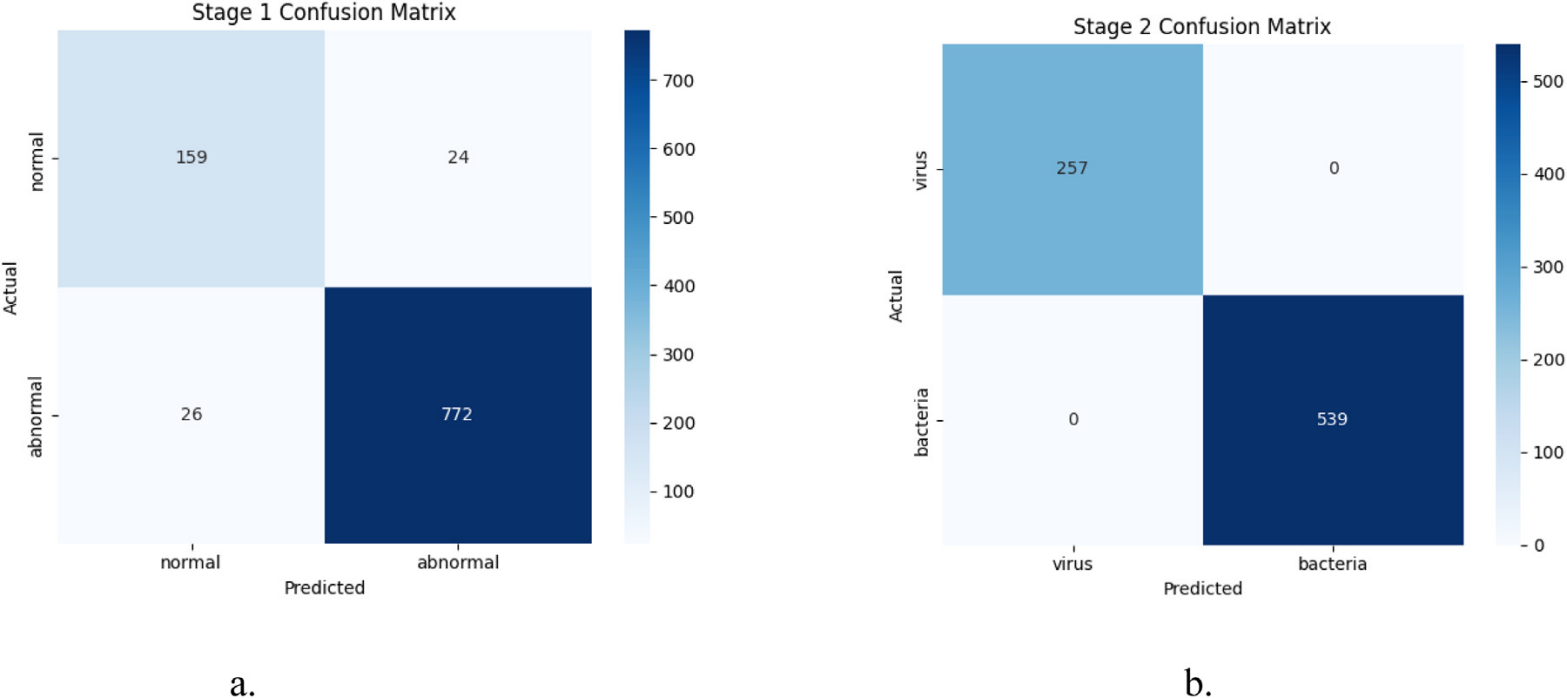
a. Confusion Matrix for Stage 1: Normal vs. Pneumonia, b. Confusion Matrix for Stage 2: Virus vs. Bacteria.

**Fig. 5:**
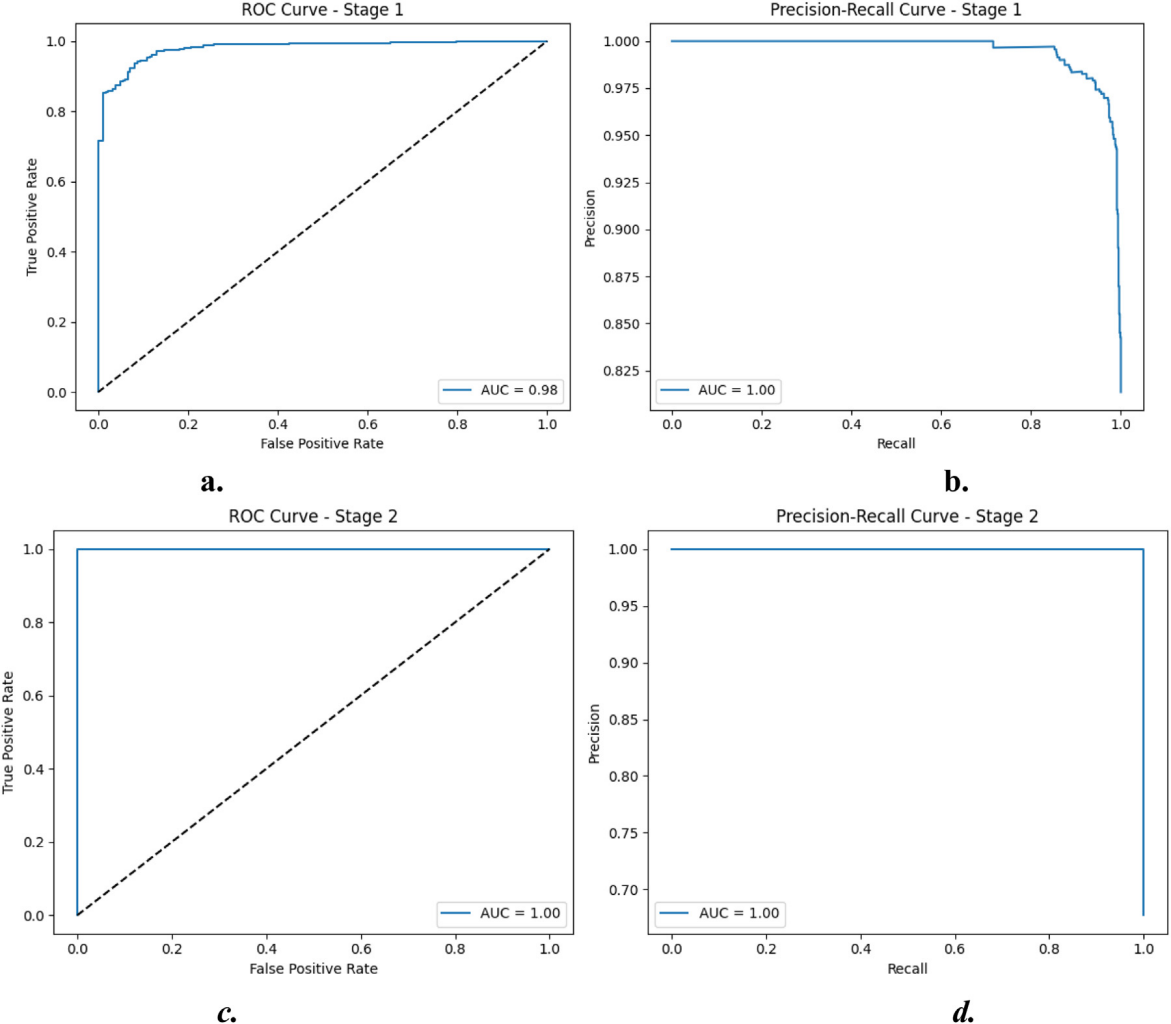
**a**. ROC Curve for Stage 1: Binary Classification, **b**. Precision-Recall Curve for Stage 1: Binary Classification, **c.** ROC Curve for Stage 2: Multi-Class Classification, **d.**Precision-Recall Curve for Stage 2: Multi-Class Classification.

**Stage 2 – Virus vs Bacteria Classification** is highlighted in Table 4. The second stage polished the classification of Pneumonia cases into *viral* and *bacterial pneumonia* using a dedicated XGBoost classifier. The model achieved **100% accuracy**, perfectly classifying all 796 pneumonia cases. Furthermore, the confusion matrix in Fig. 4a and b confirms that the two-stage ensemble model minimizes class overlap between virus and bacterial infections. See Fig. 5a–5d for ROC and PR curves

**Table 4:** Performance Metrics for Stage 2: Multi-Class Classification (Virus vs. Bacteria).

Class	Precision	Recall	F1-Score	Support
Virus	1.00	1.00	1.00	257
Bacteria	1.00	1.00	1.00	539
Macro Avg	1.00	1.00	1.00	796
Cohen’s Kappa	1.0000			
Accuracy	1.0000			
Training Time	5.34 sec			
Inference Time	0.01 sec

## Computational summary

The proposed technique demonstrates efficient processing times and inference speeds (Table 5, Table 6), making it feasible for real-time lung abnormality detection. The total processing time per image is 0.06 s, with segmentation completing in near-instantaneous time (0.00 s) due to optimized thresholding and masking techniques. Preprocessing, which includes contrast enhancement, histogram equalization, and texture preservation, takes 0.04 s, while color mapping takes 0.01 s. In terms of classification, two-stage ensemble framework, comprising normal vs. pneumonia classification followed by virus vs. bacteria executes in a total of 64.85 s. This includes both training and inference stages. Furthermore, the system maintains a minimal memory footprint (approximately 0.00 MB reported by psutil), highlighting its suitability for deployment on low-resource or edge-based medical devices.

**Table 5:** Computational Efficiency: Processing Time.

Tested Component	Time (s)
Preprocessing Time	0.04 s
Segmentation Time	0.00 s
Color Mapping Time	0.01 s
Processing Time per Image	0.06 s

**Table 6:** Computational Efficiency: Inference Speed.

Stage	Training Time (s)	Inference Time (s)
Stage 1	58.47	0.06
Stage 2	5.34	0.01
**Total Time**	**64.85 s**	

This table (Table 7: Comparison of Model Performance with Other DCNN-Based Studies) provides a comparative analysis of different pneumonia detection approaches. While the Transfer Learning with Pre-trained DCNNs study achieved the highest accuracy (98.0 %), it lacks anatomical segmentation, which is crucial for clinical explainability. The Attention-Ensemble DCNN (95.19 %) effectively emphases on important regions but is computationally expensive. Our proposed method integrates anatomical segmentation and KL divergence, improving interpretability while maintaining high performance. Future work will explore real-time optimization to balance accuracy and computational efficiency.

**Table 7:** Comparison of Model Performance with Other DCNN-Based Studies.

Study	Methodology	Models Used	Dataset Used	Accuracy (%)	Advantages	Limitations
[23]	Transfer learning with deep CNNs for pneumonia detection using chest X-ray images.	AlexNet, ResNet18, DenseNet201, SqueezeNet	5247 chest X-ray images (normal, bacterial pneumonia, viral pneumonia)	Up to 98 %	High accuracy in detecting pneumonia; effective use of transfer learning on limited datasets.	Potential overfitting due to limited dataset size; reliance on pre-trained models may limit feature learning.
[24]	Ensemble learning approach combining multiple pre-trained CNN models for pneumonia detection.	DenseNet169, MobileNetV2, Vision Transformer	Chest X-ray dataset from pediatric patients (age 1–5) at Guangzhou Women and Children’s Medical Center	93.91 %	Improved accuracy through ensemble learning; effective fine-tuning of pre-trained models.	Increased computational complexity; ensemble methods may be less interpretable.
[25]	Development of an enhanced CNN model and comparison with transfer learning models for pneumonia detection.	Enhanced CNN, VGG-19, ResNet-50, ResNet-50 with fine-tuning	Expanded Kaggle chest X-ray dataset (5863 images)	92.4 %	High accuracy with enhanced CNN; comprehensive comparison with other models.	Enhanced CNN may require more training data; potential overfitting with complex models.
**Proposed Method**	Anatomical segmentation with Deep feature extraction and 2stage classification	ASCE with Gradient Thresholding, SMOTE RESNET50, KL divergence, RF, XGBoost classification	Processed Darwin’s (Auto-Annotate) Chest X-rays(4904)	95 %	Achieves state-of-the-art accuracy, with improved explainability, interpretable predictions	Needs real-time optimization

### Cross-Dataset validation using viral pneumonia images

To evaluate generalizability outside the training dataset, we validated the proposed framework on 112 independent viral pneumonia chest X-ray images. These were treated using the ASCE pipeline for anatomical enhancement, followed by feature extraction using ResNet50. KL divergence was computed against a healthy reference distribution and appended to the 2048-D feature vector, resulting in 2049-dimensional input to the ensemble model. Of these 112 images, 94 were classified as 'virus' and 18 as 'bacteria', resulting in a classification accuracy of 83.93 %. The average KL divergence for samples predicted as virus was 11.15 ± 3.62, while bacteria predictions required an average KL of 7.63 ± 2.14. This result confirms the robustness of our KL-enhanced model in real-world scenarios and highlights its potential for deployment on unseen clinical data.

### Clinical validation through expert feedback

To evaluate the clinical importance and interpretability of the ASCE-enhanced chest X-ray images, a structured feedback form was circulated among radiologists and pulmonologists using Google Forms. The form comprised both quantitative (Likert-scale) and qualitative items related to lung segmentation accuracy, anatomical visibility, abnormality enhancement, and diagnostic support.

A total of **eight responses** were received from medical professionals with **5 to 10 years (*n*** **=** **5)** and <**5 years (*n*** **=** **3)** of experience. Their evaluations are summarized below:

• **Lung Region Segmentation Accuracy**:
  7 out of 8 respondents rated segmentation as *accurate*, while 1 marked it as *partially accurate*.
• **Anatomical Structure Visibility***(1–5 scale)*:
  Average rating: **3.62 / 5**
• **Enhancement of Abnormal Regions***(1–5 scale)*:
  Average rating: **3.75 / 5**
• **Ease of Interpretation Compared to Standard CXRs**:
  6 respondents answered *Yes*, 2 answered *Not Sure*.
• **Support for Clinical Diagnosis**:
  5 *Strongly Agreed*, 2 *Agreed*, and 1 remained *Neutral* that the method supports diagnosis.
• **Recommendation for Clinical Use**:
  All 8 respondents recommended the enhanced images as a supportive diagnostic tool.
• **Acknowledgment Consent**:
  5 experts opted to be acknowledged in the publication, while 3 preferred to remain anonymous.

These findings authenticate the proposed enhancement framework as **clinically satisfactory**, with strong consensus on its **diagnostic usefulness and interpretability**. The qualitative comments further strengthen the model’s potential as a decision-support tool in radiology. Detailed response statistics are included in **Appendix A**.

## Limitations and future work

While our **ASCE** method offers significant improvements in diagnostic imaging, it is limited in

1. **Scope of Clinical Validation**
  Although preliminary validation has been directed through structured feedback from eight practicing radiologists and pulmonologists, the method has not yet undergone **large-scale clinical trials** or deployment in real-time clinical environments.
2. **Cross-Dataset Generalization**
  Initial cross-dataset validation was directed using 112 unseen viral pneumonia cases, demonstrating promising classification accuracy and meaningful KL divergence separation. However, further validation on **diverse external datasets** remains a necessary step to assess robustness against demographic, acquisition, and labeling variability.

## Conclusion

In this study, we proposed a lightweight deep learning pipeline for enhanced lung abnormality detection using the **Anatomical Segmentation and Colour-Based Enhancement (ASCE)** technique. By integrating tissue-aware segmentation, color based visual enhancement, deep feature extraction with ResNet50, combined with KL divergence-for statistical abnormality quantification, the model effectively improves both interpretability and classification accuracy.

A novel two-stage classification pipeline was presented to first distinguish pneumonia from normal lungs and then sub-classify pneumonia cases into viral and bacterial types. Extensive experiments demonstrated:

• **95 % accuracy** in Stage 1 classification (Normal vs. Pneumonia),
• **100 % accuracy and F1-scores** in Stage 2 (Virus vs. Bacteria),
• And an **83.93 % accuracy** in cross-dataset validation on unseen viral pneumonia images.

Additionally, clinical feedback from eight medical professionals confirmed the model's visual clarity, interpretability, and practical relevance. The use of KL divergence as an support feature helped bridge the gap between statistical deviation and radiological abnormality patterns.

While initial validations are encouraging, future work will focus on broader clinical deployment, multi-center dataset evaluation, and further enhancement of segmentation quality and decision support integration. Overall, ASCE presents a promising step toward interpretable, anatomically guided AI models for medical imaging.

## Credit author statement

**Mr. Suresh Kumar Samarla:** Conceptualization, Methodology, Data curation, Formal analysis, Resources, Software Validation, Visualization, Writing –original draft, Writing-Review and editing, **Dr. P. Maragathavalli:** Supervision, Formal analysis, investigation, Writing – Review and editing

## Ethics statements

### Funding

This research did not receive any specific grant from funding agencies in the public, commercial, or not-for-profit sectors.

### Ethics approval

Not applicable, as this study does not involve human or animal subjects.

### Consent to participate

Not applicable.

### Consent to publish

All authors have read and approved the final version of the manuscript for publication.

## Declaration of competing interest

The authors declare that they have no known competing financial interests or personal relationships that could have appeared to influence the work reported in this paper.

## Appendix Supplementary materials

Image, application 1
