# Supplementary material for: An anatomically enhanced and clinically validated framework for lung abnormality classification using deep features and KL divergence
Source: MethodsX. 2025 May 14;14:103348. doi: 10.1016/j.mex.2025.103348 (PMC12141059; doi:10.1016/j.mex.2025.103348)
Supplement: Supplementary file 1 [file mmc1.docx]

**Appendix A:**

# A1:Clinical Feedback Summary

To assess the interpretability and diagnostic relevance of the ASCE-enhanced chest X-ray images, a structured Google Form was circulated among medical professionals. The form included the following items:

- Years of Experience
- Consent for Participation and Acknowledgment
- Lung Segmentation Accuracy (Yes / Partially / No)
- Anatomical Visibility Score (1–5 scale)
- Abnormal Region Enhancement Score (1–5 scale)
- Ease of Interpretation Compared to Standard CXRs (Yes / No / Not Sure)
- Support for Clinical Diagnosis (Strongly Agree to Strongly Disagree)
- Recommendation as a Diagnostic Tool (Yes / Maybe / No)

# A.2 Summary of Expert Feedback (n = 8)

The table below summarizes the feedback from eight medical experts, all of whom had between less than 5 to 10 years of clinical experience.

| **Evaluation Metric** | **Summary** |
| --- | --- |
| **Lung Segmentation Accuracy** | **7 – Yes, 1 – Partially** |
| **Mean Anatomical Visibility (1–5)** | **3.62** |
| **Mean Enhancement Quality (1–5)** | **3.75** |
| **Easier to Interpret than CXR** | **6 – Yes, 2 – Not Sure** |
| **Supports Clinical Diagnosis** | **5 – Strongly Agree, 2 – Agree, 1 – Neutral** |
| **Recommendation for Use** | **8 – Yes** |
| **Acknowledgment Consent** | **5 – Yes, 3 – No** |

Figures A1 to A5 summarize their responses. Most experts (87.5%) confirmed accurate segmentation. (Figure A1), considered the enhanced images easier to interpret than traditional CXRs (Figure A2), and 87.5% agreed that the enhancement improves diagnostic support (Figure A3). All respondents recommended ASCE-enhanced images as a useful diagnostic aid (Figure A4), and the majority provided consent for acknowledgment in this research (Figure A5). These results confirm the clinical interpretability, usability, and acceptability of the proposed ASCE framework from a practitioner’s perspective.

# A3: Expert Comments Summary

In addition to the structured form responses, participants were inspired to provide open-text feedback. The following selected comments reflect the clinical perspectives and potential use cases of the ASCE-enhanced chest X-ray images:

- *“ASCE images are easier to interpret than raw X-rays in moderate pneumonia.”*
- *“I’d still prefer to refer to the original for early-stage cases.”*
- *“The enhancement might be helpful for training junior doctors.”*

These qualitative understandings reinforce the usefulness of ASCE in facilitating diagnosis, particularly in moderate cases where abnormalities are more visually deceptive. The responses also underscore the need for flexibility in clinical backgrounds preserving access to both original and enhanced views especially for nuanced or early-stage findings. Moreover, the educational value of the enhancements for junior clinicians and trainees was positively noted.


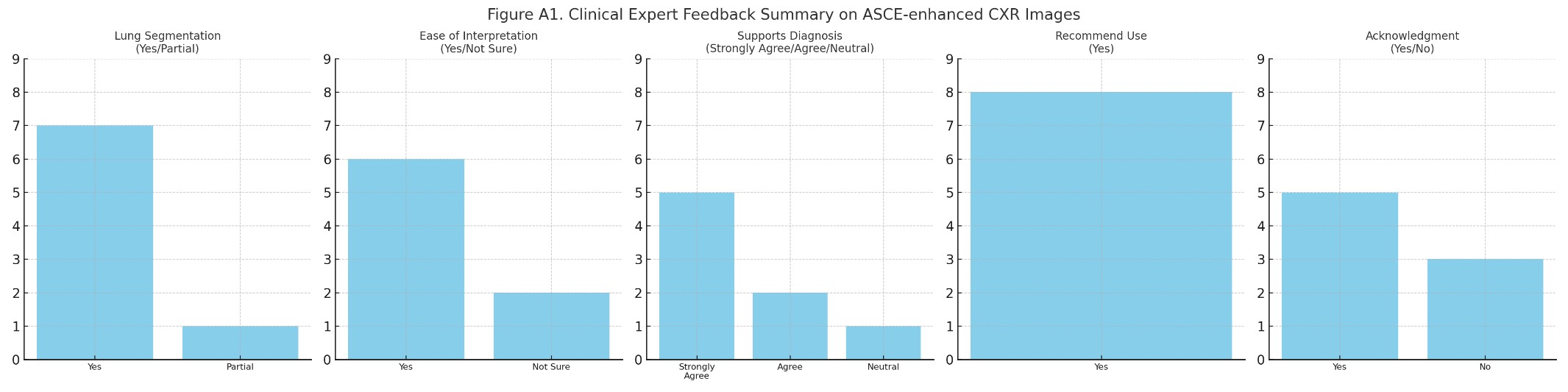


*Figure A1. Lung segmentation accuracy as rated by 8 medical professionals.*

**a**

**b**

**c**

**d**


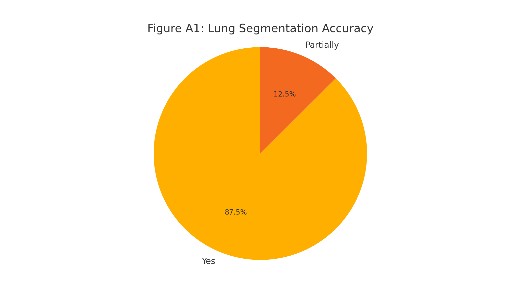

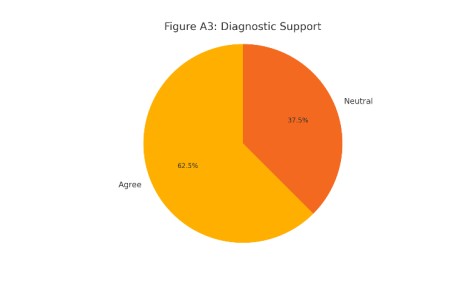

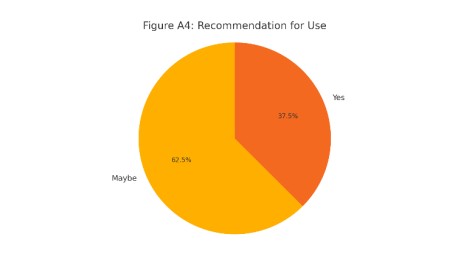

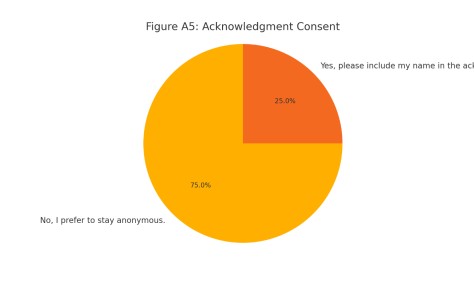


1. *Figure A2. Expert opinion on ease of interpretation compared to standard CXR.*
2. *Figure A3 Expert Ratings on Diagnostic Support Provided by ASCE-Enhanced Images.*
3. *Figure A4 Clinician Willingness to Recommend ASCE-Enhanced Images for Clinical Use.*
4. *Figure A5 Consent to Be Acknowledged in the Research Publication.*
